# Membrane Antigen Targeting in Acute Myeloid Leukemia Using Antibodies or CAR-T Cells

**DOI:** 10.3390/cancers16213627

**Published:** 2024-10-28

**Authors:** Ugo Testa, Germana Castelli, Elvira Pelosi

**Affiliations:** Department of Oncology, Istituto Superiore di Sanità, Viale Regina Elena 299, 00161 Rome, Italy; germana.castelli@iss.it (G.C.); elvira.pelosi@iss.it (E.P.)

**Keywords:** immunotherapy, chimeric antigen receptor T cells, acute myeloid leukemia, monoclonal antibodies, bispecific antibodies, membrane antigens, immunologic profile

## Abstract

This review explores the emerging area of the therapeutic use of antibodies and chimeric antigen receptor (CAR)-T cells for the treatment of acute myeloid leukemia (AML). Through a detailed analysis of the existing literature, this paper highlights the different categories of AML antigens for immunotherapeutic targeting, the most recent applications on antibodies, including bispecific immune cell engagers and CAR-T cells, to the therapy of patients with refractory/relapsing AML The studies performed in AML patients using BisAbs and CAR-T cells have shown that only a limited number of AML patients show sustained responses to these therapies, thus underlying AML heterogeneity as a major challenge. Several studies have addressed the potential mechanisms underlying the resistance of AMLs to antibody-directed immunotherapies. A better understanding of the barriers hampering the successful development of AML immunotherapy is required. However, in spite of the limitations, the studies recently carried out have shown the peculiar sensitivity of some AML subtypes to immunotherapy and have provided the basis for future studies, such as multiplex antigen targeting, which hold the promise of successful development.

## Simple Summary

This review explores the emerging area of the therapeutic use of antibodies and chimeric antigen receptor (CAR)-T cells in acute myeloid leukemia (AML). Through a detailed analysis of the existing literature, this paper highlights the most recent applications of antibodies, including bispecific immune cell engagers and CAR-T cells, to the therapy of relapsing/refractory AML. Furthermore, it discusses the potential mechanisms underlying sensitivity or resistance to antibody- and CAR-T cell-based therapies. Overall, this review underscores the difficulties and potentialities of antibody-guided immunotherapies in the treatment of AML patients.

## 1. Introduction

Acute myeloid leukemia (AML) is a heterogeneous group of diseases, characterized by the proliferation and accumulation of leukemic cells blocked at an early stage of myeloid cell differentiation. At the cellular level, AMLs are organized as cellular hierarchies originating from cells endowed with the capacity to initiate and maintain leukemic cells and are functionally defined as leukemia stem cells (LSCs). At the molecular level, AMLs derive from mutagenic events occurring in hematopoietic stem cells (HSCs) and/or hematopoietic progenitor cells (HPCs) through a multistep process involving the acquisition of recurrent driver gene mutations and/or chromosome translocations.

Numerous studies have contributed to the characterization of molecular abnormalities underlying AMLs with the identification of recurrent chromosomal aberrations, gene mutations, and gene translocations, allowing the classification of this heterogeneous group of leukemias into various molecular subgroups characterized by different genetic alterations and responses to current treatments [1,2,3,4,5].

The most recent molecular characterization of adult AMLs reported the definition of 16 molecular classes including 100% of the 3653 AML patients analyzed [1]. In this classification, AMLs corresponding to acute promyelocytic leukemia (APL) were excluded (1–2% of the AMLs); APL is a distinct subtype of AML characterized by t(15;17)(q22;q21), a balanced reciprocal translocation involving *PML* and *RARA* genes. In this classification, each class corresponds to diverse biological AML subgroups, associated with different clinical presentations, probabilities of response to induction chemotherapy, risks of relapse, and death over time [6].

Some molecular classes were defined by cytogenetic alterations and included patients with complex cytogenetic alterations and translocations (Table 1):

- Patients with complex karyotypes (CKs) formed a group of 10.3% of AMLs and were characterized by ≥3 unbalanced cytogenetic alterations, frequent *TP53* mutations (65%), a paucity of other somatic mutations and were usually observed in older patients and were associated with poor outcomes [1].
- A second subgroup was characterized by <3 trisomies and frequently involved +8, +11, +13, +21, and +23 but not deletions (about 11% of total AMLs); this group displays infrequent *TP53* alterations (4%) and is associated with a more favorable prognosis [6].
- Other groups are characterized by chromosomal translocations: The inv(16)(p13q22) and the less frequent t(16;16)(p13q22) determine the formation of a chimeric gene *CBFB-MY11* consisting of the 5′ portion of the smooth muscle myosin heavy chain gene (MYH11), characterizing 4–5% of total AMLs, in association with a favorable prognosis;
- t(8;21)(q22;q22.1) determines the formation of *RUNX1-RUNX1T1* fusion, characterizing 5–6% of AMLs, associated with favorable or intermediate prognosis;
- AMLs characterized by rearrangement of the *KMT2A* gene [*KMT2A_MLLT3* fusion caused by t(9;11)(q21.3;q23.3) is the most frequent] represent 3–4% of adult AMLs and are associated with intermediate prognosis;
- The t(6;9)(q23;q34) translocation, observed in about 1% of adult AMLs, generates the *DEK/NUP214* fusion gene, causes an aggressive disease with poor prognosis; AML with inv(83) or t(3;3) is an extremely aggressive and rare AML subtype (0.5–1% of AMLs) related to the juxtaposition of the *GATA2* gene enhancer in proximity to the *MECOM/EVI1* promoter [6].
- Other AML molecular subtypes are characterized by some recurrent mutations:
- A large cluster of AMLs (>28% of AMLs) is characterized by the presence of secondary AML (sAML) type mutations, such as *SRFSF2, SF3B1, U2AF1, ZRSFR2, ASXL1, EZH2, BCOR, STATG2, RUNX1, SETBP1*, and *MLL*^PTD^ mutations; patients in this cluster were older and displayed a higher incidence of antecedent hematologic diseases compared to the rest of AML patients [1]. These patients were subdivided into two groups, sAML1 (secondary AML1, about 5% of total AMLs) with a single-class-defining mutation and sAML2 (about 24% of total AMLs) with ≥2 class-defining mutations; sAML1 patients had a better prognosis than sAML2 patients [6].
- A group of AMLs, corresponding to about 2% of adult AMLs, is characterized by biallelic mutations of the *CEBPA* gene and is associated with favorable prognosis; particularly, bZIP in-frame insertions/deletions are associated with good prognosis [6].
- The largest group of AMLs is characterized by mutations of the nucleophosmin 1 gene (*NPM1)* gene (30–35% of total AMLs), is associated with a normal karyotype, and usually occurs in de novo AMLs; this is a clinically heterogeneous group associated with variable prognosis, related to variability of co-associated mutations [6].
- A rare group of AMLs (<1%) is characterized by the presence of *DNMT3A/IDH1-2* mutations, in the absence of other mutations, and is associated with intermediate/poor outcomes [1]. A total of 6% of patients, not clustering with any AML molecular class, were defined as Molecularly Not Otherwise Specified. Finally, about 2% of patients had no identifiable mutation [6].

This molecular classification, together with clinical and other diagnostic criteria, allows a classification of AML patients and provides a tool for the identification of their optimal therapeutic strategy.

The basic treatment of AML patients involves an induction chemotherapy regimen (7 + 3 regimen), followed by consolidation chemotherapy and/or allogeneic hematopoietic stem cell transplantation (allo-HSCT) for patients with a high risk of relapse: For older or unfit patients, an alternative treatment is usually adopted based on the administration of azacitidine or hypomethylating agents, in combination with Venetoclax, targeting the anti-apoptotic protein Bcl-2. The definition of the genetic landscape of AMLs has led to the identification of several driver genes that can be specifically targeted using specific inhibitors and that can be used in relapsed/refractory (R/R) or newly diagnosed AML patients.

However, in spite of the development of this therapeutic armamentarium, a consistent proportion of AML patients relapse or are refractory to this treatment, and their prognosis is poor. The only potentially curative approach for these patients is represented by allo-HSCT; however, allo-HSCT is limited only to a part of these patients, and its outcome is greatly conditioned by the achievement of a condition of remission, with a minimal residual disease (MRD) negativity before transplantation [7].

AML is a valuable therapeutic target of immunotherapy. This conclusion is strongly supported by the documentation of the graft versus leukemia (GVL) effect of the donor bone marrow patients undergoing allo-HSCT, an effect mainly mediated by donor T cells [8,9]. Therefore, there is a strong rationale to develop immunotherapeutic approaches for the treatment of AML patients. Various types of immunotherapeutic approaches have been developed for the treatment of AMLs: Some of these approaches were based on a strategy aiming to stimulate an endogenous anti-AML immune response, while other approaches were based on the immunological targeting of antigens specifically or preferentially expressed on the membrane of leukemic cells, compared to normal hematopoietic cells [10,11]. However, the development of effective immunotherapy is hampered by challenges limiting its effectiveness. First, AML is a highly heterogeneous disease at the genetic and epigenetic level; the bone marrow microenvironment in AML patients is basically immunosuppressive and protects leukemic stem cells from immune-mediated destruction; AML cells have developed mechanisms for immune escape, such as reduction or loss of HLA expression [12]. Transcriptomic studies have contributed to better defining the bone marrow immune microenvironment, showing an inferior survival in AMLs with signatures of immune depletion or enrichment of senescent-like CD8^+^/T cells and association of mutated *TP53* status with increased infiltration with cytotoxic T cells [13,14,15]. A better understanding of the AML immune microenvironment and the role of inflammation in AML is fundamental for the identification of AML subsets more sensitive to immunotherapy [16,17].

The present review will analyze recent developments in the immunotherapies of AML based on agents that target membrane antigens preferentially or more selectively expressed on AML cells.

## 2. Experimental and Clinical Studies of AML Immunotherapy Through Targeting of Membrane Antigens

A consistent number of studies of immunotherapy of AML was carried out through the targeting of membrane antigens preferentially expressed on the surface of AML cells (Table 2).

**Figure 1:**
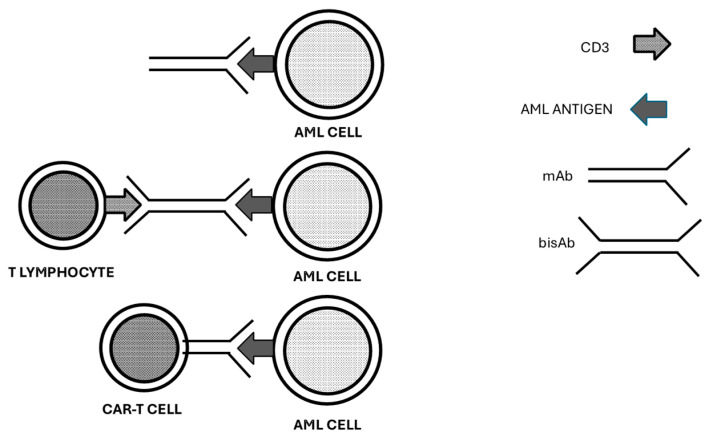
Immunotherapeutic targeting of AML cells using monoclonal antibodies, bispecific antibodies, or CAR-T cells.

Targeting of AML membrane antigens was carried out using three main types of antibody-mediated strategies: One strategy involves the use of simple monoclonal antibodies specifically targeting a membrane antigen, bispecific monoclonal antibodies targeting both a specific membrane antigen on AML cells and CD3 on T cells, or CAR-T cells, T lymphocytes genetically engineered to target a specific membrane antigen present on AML cells (Figure 1).

### 2.1. Targeting of CD38 in AML

CD38 is widely expressed in immune cells, and its level of expression is dependent on cell maturation and activation. CD38 is a transmembrane glycoprotein that can function as an adhesion partner for CD31 or a multifunctional ectoenzyme involved in the catabolism of NAD^+^ and NADP. Clinical experience with the monoclonal antibody anti-CD38 Duratumumab has demonstrated consistent clinical efficacy in multiple myeloma, with a favorable on-target/off-tumor toxicity profile. Preclinical studies have supported CD38 as a valuable therapeutic target for AML for its expression observed in about 70% of cases [18]. The targeting of CD38 on AML blasts with a blocking anti-CD38 mAb not only facilitates their removal by immune cells but inhibits also leukemic blast metabolic capacity by blocking mitochondrial transfer from mesenchymal stromal cells to leukemic cells [19]. Furthermore, in suitable preclinical models, the Duratumumab combination treatment with Venetoclax resulted in enhanced antileukemic activity [20].

The majority of bulk AML cells highly express CD38, and this provides a rationale for its pharmacologic targeting. The phase II NCT 03067571 clinical trial is evaluating Duratumumab in the treatment of patients with relapsed or refractory AML or high-risk MDS; however, the results of this study are not yet available.

The study of individual AML patients with high-risk disease or with relapsing/refractory (R/R) disease supported the rationality of CD38 targeted therapy in association with traditional chemotherapy or demethylating agents and Venetoclax [21]. However, preclinical models have shown that CD38 inhibition induced antibody-dependent phagocytosis and interference with AML cell trafficking but lacked a pronounced antileukemic activity [22].

CD38 expression is lower in the fraction of cells endowed with properties of leukemic stem cells, cells involved in the generation, maintenance, and relapse of AML, a finding potentially limiting the therapeutic efficacy of CD38 targeting. Murtheda and coworkers showed that CD38 expression on leukemic blasts is significantly increased by interferon-gamma (IFN-γ) [23]. Since IFN-γ is produced during T cell activation, this finding triggered the development of a bispecific antibody capable of both targeting CD38 and activating T-lymphocytes; to this end, it generated a single-chain bispecific antibody CD38-CD3 BIONIC (BN-CD38) by inserting a CD38 nanobody between the light and the heavy chains on the anti-CD3 antibody; BN-CD38 exerted a potent antileukemic activity in patient-derived xenograft models, including those originated from leukemias containing a high fraction of CD34^+^/CD38^−^ cells [23].

A recent study reported the first results of a phase I clinical study based on the administration of XmAb 18968, a novel CD38-CD3 bispecific T cell engager with an Fc domain modified to minimize Fcγ receptor binding and non-selective T cell activation, resulting in reduced cytokine release, to 12 adult R/R AML patients [24]. The drug was administered at four dose levels (from 0.8mg to 1.5 mg). Two of the treated patients achieved a CR- MRD-negative and proceeded to allogeneic HSCT. In the whole group of patients, the mOS was 6.2 months [24].

Martin-Sanchez et al. have reported the development of a trispecific T cell engager (CD38xCD3xCD28) and have explored its potential use in the treatment of older AML patients [25]. First, these authors characterized CD38 expression in 241 samples of older AML patients and observed that 55% of cases displayed a homogeneous CD38 expression on leukemic blasts, while in about 44% of cases, this expression was heterogeneous [25]. Preclinical studies on primary BM AML samples supported the antileukemic activity of the trispecific TCE, with limited on-target off-tumor toxicity [25].

Preclinical studies have shown that CAR-T cells targeting CD38 are effective against models of AML, as well as other hematologic malignancies, such as T-ALL and multiple myeloma [26]. In xenograft models of human hematopoiesis, CD38-CAR-T cells resulted in an expected reduction in hematopoietic progenitors, thus suggesting some caution when translating CD38-CAR-T cell immunotherapy into the clinic [26]. Few studies have explored the safety and efficacy of CD38-directed CAR-T cells in R/R AML patients. Thus, Cui et al. explored CD38-CAR-T cells in six AML patients relapsing after allo-HSCT; four of six patients achieved a CR 4 weeks after CAR-T cell therapy; the mOS was 7.9 months, and at 6 months, the relapse rate was 50% [27]. All six patients experienced manageable side-toxic effects [27].

### 2.2. Targeting of CD33 in AML

CD33 is the typical example of a membrane antigen preferentially expressed on leukemic cells compared to the normal myeloid counterpart. The value of CD33 as a therapeutic target for AML is suggested by the clinical studies carried out with Gemtuzumab-Ozagamicin (GO), a humanized anti-CD33 monoclonal antibody conjugated to a highly toxic natural product calicheamicin that induces double-strand DNA cleavage. The fractionated administration of GO was approved by the FDA in 2013 alone or with chemotherapy for de novo AML and as a single agent for R/R CD33^+^ AML. A recent randomized clinical trial confirmed the efficacy of GO when added to chemotherapy in improving the outcomes of *NPM1*-mutant AML patients [28]. Furthermore, a meta-analysis evaluating a consistent number of clinical studies provided evidence that the addition of GO to standard chemotherapy improves relapse-free survival and overall survival in the group of AML patients with a favorable or intermediate cytogenetic risk [29].

Several bispecific anti-CD33 antibodies have been developed and evaluated in phase I clinical studies. Thus, a first study evaluated AMG330, a bispecific T cell engager that binds CD33 on leukemic cells and CD3 on T cells; the patients were treated with continuous intravenous infusion (0.5 ug/day–1.6 mg/day) on 14-day or 28-day cycles, and 10% of patients experienced grade 3–4 CRS [14]. Eight patients achieved a CR; of the 52 non-responders, 37% had >50% reduction in AML blasts [30].

AMG673 is a second BiTE with an extended half-life based on a construct that binds both CD33 and CD3 and is genetically fused to the N-terminus of a single-chain IgG Fc region [31]. This compound was evaluated in the context of a phase I clinical study in 27 R/R AML patients showing serious adverse events in 37% of cases and a limited antileukemic efficacy (with 44% of patients showing a blast reduction, 22% >50%, and 1 CR) [31].

AMV564 is a tetravalent tandem diabody with two avidity recognition sites for CD33 and two for the invariant CD3-epsilon chain; this compound is smaller than immunoglobulins and should penetrate better into tissues, with a half-life of 18–23 h, and could be conceivably administered via daily injections. Interestingly, studies in experimental models have shown the capacity of AMV564 not only to induce clearance of CD33^+^ leukemic cells but also to generate an immune memory in vivo [32]. A phase I clinical study (NCT03144245) evaluated 36 R/R AML patients (mean age 71 years) with growing doses of AMV564 (from 0.5 ug to 300 ug/day); no dose-limiting toxicities were reported; no grade 3 or 4 CRS were observed [33]. Bone marrow blast reductions were observed in 49% of patients; two patients achieved a CR and one a PR [33].

In order to improve the efficacy of bispecific CD33 antibodies, a bispecific antibody targeting both CD33 and CD123 was developed: Thus, a dual targeting CD33/CD123 NANOBODY T cell engager was produced [34]. This CD33/CD123-TCE was able to kill both single- and double-positive AML cells, outperforming single-targeting TCE in a subset of primary AML samples; furthermore, CD33/CD123-TCE markedly inhibited leukemic cells in a mouse model and target cells in nonhuman primates without signs of cytokine release [34]. The CD33/CD123-TCE holds the promise of improving the therapeutic window by increasing the capacity to kill heterogeneous AML clones, with a safer safety profile compared to first-generation TCE [34].

Recent studies have addressed a considerable interest in the investigation of CD33-targeted CAR-T cells in the treatment of R/R AMLs (Table 3). These studies have explored also the potential toxicities related to CD33 targeting by CAR-T cells at the level of normal HPCs/HSCs. In this context, particularly interesting was a preclinical study based on the development of CAR-T cells using an anti-CD33 scFv recognizing the membrane-proximal C2-set domain of CD33 and containing a DARIC receptor, conferring responsiveness to low doses of rapamycin; rapamycin withdrawal paused CAR-T cell activation [35]. DARIC22 T cells exhibited potent antileukemic activity against CD33-positive targets but spared normal CD34^+^ HSCs [35]. Studies in xenograft models showed the reversibility of CAR-T cell activation using DARIC33 CAR-T cells [35]. Based on these observations, the phase I PLAT-08 trial was initiated, supporting the feasibility of rapamycin activation of DARIC33 CAR-T cells [35]. Another two recent studies have supported the rationale of developing CAR-T cells targeting the membrane-proximal C2-set domain of CD33 [36,37]. Compared with CAR-T cells targeting the membrane distal domain of CD33, CAR-T cells targeting proximal CD33 showed greater in vitro and in vivo efficacy against primary CD33-positive AML cells, including those with low density of expression, without increased expression of exhaustion markers [36,37].

Initial studies by Kenderian et al. have shown that CD33-directed CAR-T cells exert potent antileukemic activity against human AMLs; however, xenograft models of hematopoietic toxicity also showed human cytopenias and a consistent reduction in human hematopoietic progenitors; to bypass the possible negative effects derived from systemic myelosuppression, a strategy based on transient expression of anti-CD33 CAR by RNA modification was developed [38].

A single-arm phase I clinical trial (NCT 031268649) explored the safety and efficacy of CD33-specific CAR-T cells in R/R AML patients [37]. Ten patients were enrolled, but only three were infused at the lowest dose level (0.3 × 10^6^ cells/Kg); no dose-limiting toxicities were observed, and two patients had CRS grade 2 or 3; however, no significant response to treatment was observed [39].

Preclinical studies were also focused on defining the optimal CD33 CAR construct for the final CD33-CAR-T preparation. Thus, Qin et al. have reported preclinical development of second-generation CD33-targeted CAR-T cells; the analysis of the various CD33 CAR constructs has identified the CD28/CD3ζ CD33 CAR construct containing a lintizumab-derived scFv as the optimal one and has supported its clinical evaluation in children with R/R AML [40]. Using these CAR-T cells, Shah et al. have recently reported the results observed in 19 children/young AML patients, treated at four different dose levels: DL1 3 × 10^5^/Kg, DL2 1 × 10^6^/Kg, DL3 3 × 10^6^/Kg, and DL4 1 × 10^7^/Kg [41]. CR was observed only in two patients treated at the highest dose level, associated with an MRD-negative status and with myeloid aplasia: One of these two patients proceeded to allogeneic HSCT, while the other patient was not transplanted and spontaneously recovered from aplasia and remained in remission after 119 days [41]. Based on these observations, enrollment continued at DL4.

Pan et al. have recently reported the preliminary results of a phase I clinical study (NCT 04835519) involving four R/R AML patients treated with 5 × 10^5^/Kg CD33-CAR-T cells; these CAR-T cells were functionally enhanced by the addition of a potentiating molecule linked to human CD33 scFv [42]. One patient experienced grade 4 CRS, while the other three patients exhibited grade 1–2 CRS [42]. Two patients achieved CR and were MRD-negative at day 30, while the remaining two patients did not respond; one of these patients remained disease-free for over two years [42]. All patients displayed some depletion of normal CD33^+^ cells [42].

Zuo and coworkers have explored the mechanisms responsible for the unsatisfactory effectiveness of CD33-CAR-T cells in AML patients [43]. The study showed that CAR-T cells are less effective at killing myeloid leukemia than B-lineage leukemia, and these defects are caused by impaired antigen downstream signaling, leading to impaired anti-tumor function [43]. Particularly, CAR-T cells exposed to myeloid cells display impaired calcium, ZAP70, ERK, and c-JUN signaling, and these defects are in part caused by high CD155 expression by AML cells [43]. Overexpression of c-JUN but not of other antigens downstream signaling components restores an efficient cytolytic activity of CAR-T cells against AML cells [41]. C-JUN overexpression increases co-stimulatory molecules and cytokines through potentiation of ERK or transcriptional activation [43]. These observations have supported the development of a phase I clinical trial (NCT 03835519) involving the enrollment of four initial R/R AML patients with a median age of 9.5 years and who received 2–6 lines of prior therapy; the patients received a single dose (0.5 × 10^6^ cells/Kg) overexpressing c-JUN [43]. Three patients experienced CRS of grade 1–2 and one patient of grade 4; two patients had grade 1 neurotoxicity; two patients developed grade-1 GVHD [25]. Two patients achieved a CR with MRD-negative status, with incomplete hematologic recovery; one patient had a partial response, with a reduction in blast cell counts; one patient did not respond to treatment [43]. The two patients achieving a CR were transplanted and remained disease-free and alive for more than two years [41]. These observations show that overexpression of c-JUN has the potential to restore the effective anti-AML response of CD33-CAR-T cells [43].

Bispecific CAR-T can be produced through different procedures, allowing the generation of four different types: (i) pooled CAR-T, where different clones are generated, each with its specific CAR and its targeting capacities; (ii) compound CAR-T, where two CARs, each with its co-stimulatory and activating domains, are expressed by the same cell; (iii) split CAR-T, where two different CARs (a main CAR and a co-stimulatory CAR) are linked differentially to co-stimulatory or activating domains and are expressed in the same cell; (iv) tandem CAR-T, where two different CARs are linked to the same co-stimulatory and activating domains.

Preclinical studies have supported the pronounced antileukemic activity of CD33-CLL1 compound CAR-T cells in both in vitro and in vivo mouse xenograft models [44]. Using these CAR-T cells, Liu et al. have treated R/R AML patients and reported the case of a 6-year R/R AML patient achieving CR and proceeding to allo-HSCT [44]. Recently, the generation of tandem CAR-T cells exhibiting potent antileukemic activity and low cytotoxicity towards normal HPCs/HSCs was reported, thus supporting their clinical evaluation in future studies [45].

Interestingly, a recent study reported the development of CD33/CD123 bispecific CAR-T cells able to control AML cells in xenograft models of AML, showing no on-target off-tumor effects, thus differentiating between leukemic and normal cells, at variance with CAR-T cells targeted at either CD33 or CD123 [46]. These observations support clinical investigation based on human CD33/CD123 bispecific CAR-T cells.

A recent study suggested a new complex approach to avoid the myelotoxicity elicited by CD33-targeting agents based on the concomitant infusion of knock-outed HSCs and CD33-targeting agents [47,48]. Preclinical studies have shown that human HSCs CD33-ablated using genomic engineering methods (CRISPR/Cas9 technology) are able to engraft and repopulate a functional multineage hematopoietic system in vivo [47,48]. CD33-edited hematopoietic cells are protected from the myelotoxic effects of CD33-targeted therapeutics, including CD33-CAR-T cells [49,50]. The VBP01 (NCT 04849910) clinical trial provided preliminary evidence in three CD33^+^ R/R AML patients that CD33-deleted HSCs and HPCs display normal engraftment and repopulation after allogeneic HSCT and tolerate post-transplantation GO without cytopenia [51]. A more recent study reported the evaluation of the first six treated patients, all exhibiting successful engraftment with CD33-negative cells, displaying minimal cytopenias following GO treatment [52]. The results of this study must be confirmed in a larger number of patients, with a long-term evaluation of the reconstituted CD33-negative hematopoietic system.

In addition to T cells, NK cells represent another source of immune cells that can be engineered to target CD33 antigen. NK cells offer, with respect to T cells, the advantage of not inducing graft versus host disease, and to induce fewer side effects and hold an intrinsic CAR-independent killing capacity against AML but possess a more limited in vivo lifetime compared to T cells. Albinger et al. reported the generation of CAR-targeted, CAR-modified NK cells engineered to target CD33-expressing cells [51,52]. Huang et al. reported the results of a phase I study enrolling 10 adult R/R AML patients treated with three infusions of CAR-NK CD33-targeted cells (6 × 10^8^ or 1.2 × 10^9^ or 1.8 × 10^9^ cells) [53]. The treatment was well tolerated with one patient reporting CRS grade 1 and one patient CRS grade 2 [51]. Six of ten patients achieved CR, MRD^-^ on day 28 of the assessment [53].

The activity of CAR-NK cells is often inhibited by the interaction between HLA-E expressed on leukemic cells and the NK group 2A (NKG2A) receptor encoded by the *KLRC1* gene and expressed on NK cells [54]. To improve the fitness and to reduce the risk of exhaustion of CAR-NK cells, Bexte et al. have generated CD33-specific CAR-NK cells combined with CASPR/Cas-9-based gene disruption of *KLRC1* gene encoding NKG2A; the CAR-NK-CD33-KLRC1^KO^ cells thus generated exhibited potent antileukemic killing of CD33-positive leukemic cells [54].

### 2.3. Targeting of CLL1 in AML

CLL1 (also known as CLEC12A) is a membrane antigen expressed on leukemic stem cells and AML blasts but not on normal hematopoietic stem cells and therefore represents a potential therapeutic target for leukemia immunotherapy. Preclinical studies have shown that MCLA-11F, a BiTE targeting CLL1 on leukemic cells and CD3 on T cells, induces T cell activation, proliferation, pro-inflammatory cytokine release and redirects T cells to lyse CLL1-positive leukemic cells [55]. In vitro studies using primary AML samples showed that MCLA-117 induced a marked AML blast cell killing [55]. MCLA-117 was evaluated in the context of a rump-up phase I clinical study in 58 R/R AML patients across 11 different dose levels [56]. The treatment was well-tolerated, with no dose-limiting toxicity events and with CRS events, mostly of grade 1–2, observed in 21 patients [38]. The clinical responses were limited with six patients exhibiting a >50% blast cell count decrease, four patients with a >70% blast cell count decrease, and one patient achieving a leukemia-free morphological state [56].

A recent study compared two CLL1xCD3 bispecific antibodies with different formats, that is, 2 + 1 and 1 + 1 with respect to CD3 binding affinity, efficacy, and basal cytokine release [57]. The ABL603 2 + 1 BiTE showed a limited binding in the absence of CLL1, but in spite of this attenuated CD3 binding capacity, it exhibited much stronger T cell activation and anti-tumor activity compared to a 1 + 1 BiTE [57]. These observations suggest that ABL602 2 + 1 could represent a new therapeutic option for treating AML.

A recent phase I study reported the preliminary results relative to the treatment of seven R/R AML patients with the bispecific CLL1/CD3 QLF32101 antibody, showing high affinity for CLL1 and low affinity for CD3; 5/7 patients displayed a significant reduction in blast cell counts [58].

Several studies have explored the safety and efficacy of CLL1 targeting CAR-T cells both in pediatric and adult AMLs. Zhang et al. reported the preliminary results of a phase I/II clinical study including four pediatric R/R AML patients infused with anti-CLL1 CAR-T cells generated through transduction of a lentiviral vector 4SCAR (CLL1-CD28-CD27-ζ-CAR-T); the patients received a single infusion of CAR-T cells after lymphodepletion [59]. No major adverse events related to the treatment were observed. A total of 75% of the treated patients achieved complete remission and MRD negativity; the other patients remained alive for 5 months [59]. One of these four patients was bridged to allo-HSCT [59]. In a second study, the same authors compared autologous T cells expressing CLL1-CD28-CD27-ζ-CAR-T cells and the inducible caspase 9 suicide gene (four patients) or CLL1-41BB-ζ-CAR-T cells (three patients) in a total of seven pediatric AML patients [60]. CAR-T cell infusions were well-tolerated, and five patients achieved a CR (3 MRD-negative and 2 MRD-positive), with a 1-year OS of 57% [60].

Zhao and coworkers have reported the safety and the efficacy of CAR-T cell therapy targeting CLL1 in 47 adult R/R AML patients (17 refractory and 30 relapsing), subdivided into two subgroups according to the presence or not of extramedullary disease (EMD). Among the 20 patients with EMD and the 27 without EMD, complete remission in bone marrow was achieved in 65% and 81% of patients, respectively; although the overall survival, progression-free survival, and duration of remission were shorter for patients with EMD compared to those without EMD, this difference was not statistically significant [61].

Another phase I clinical study evaluated CLL1-41BB-ζCAR-T cells in eight children R/R AML patients exhibiting high CLL1 positivity [62]. The patients were treated with a single infusion of 0.35 × 10^6^ CAR-T cells/Kg after lymphodepletion with fludarabine/cyclophosphamide [62]. The treatment was well-tolerated, with grade 1–2 CRS observed in all treated patients [62]. Concerning the response to treatment, five patients achieved a morphological leukemia-free state (MLFS) (MRD-negative in four cases and MRD-positive in one case; 1PR and 1 SD) [62].

A recent study reported the multiple targeting of both CLL1 and CD33 in nine R/R pediatric AML patients [49]. These patients received escalating doses (1 to 3 × 10^6^ cells/Kg) of CAR-T cells expressing CLL1 and CD33 CAR as single or split doses after lymphodepletion with fludarabine/cyclophosphamide [63]. A total of 7/9 patients achieved an MRD-negative (by flow cytometry) complete remission; six of the seven responding proceeded to HSCT; two patients were not responsive, and one of them displayed an AML-only CD33-positive [63].

Other studies have evaluated CLL1-CAR-T cells in adult AML patients (Table 4). Thus, Jin has evaluated CLL1-41BB-ζ-CAR-T cells in 10 adult R/R AML patients (ChiCTR20000041054) [64]. These patients were treated with a single infusion of 1 or 2 × 10^6^ CAR-T cells/Kg; all patients developed a CRS of grade 3 and 2 of grade 4; the CR-Cri rate was 70%, and at a median follow-up of 173 days, six patients were alive [64]. A more recent report on this trial updated the results to 30 patients treated at four different dose levels [65]. All patients experienced CRS of grade 0–2 in 18 patients, grade 3 in 11 patients, and grade 4 in 1 patient; all patients experienced hematological toxicity, with grade 3–4 granulocytopenia, anemia, or thrombocytopenia observed in most of the patients [65]. Patients who received haploidentical bridge transplantation after CAR-T cell therapy resolved hematological toxicities [65]. A total of 73% of patients responded to treatment, with 40% of CR/MRD-negative and 33% of CR/MRD-positive cases [65]. Allo-HSCT post-CAR-T cell therapy improved overall survival, and two transplanted patients survived >25 months post-therapy [65].

Two recent studies have explored the antileukemic activity of new CARs targeting CLL1. Thus, Xie et al. have developed bicistronic CAR-T cells targeting CLL1 and CD123; interestingly, these CAR-T cells were generated also including the marker/suicide gene RQR8, allowing the clearance of CAR-T cells if necessary [66]. In vitro studies have supported a marked capacity of CLL1-CD123-CAR-T cells to induce the killing of leukemic cells and to reduce the risk of antigen escape [66]. Another recent study reported the development of an allogeneic anti-CLL1 CAR-T cell therapy (CB-012). CB-012 CAR-T cells expressed a fully human anti-CLL1 scFv-containing CAR construct and exhibited potent antigen-dependent cytotoxicity against AML cells; in xenograft AML models, a single infusion of CB-012 cells exerted a marked antileukemic activity [53]. These observations support the evaluation of CB-012 cells in the context of a phase I clinical study in R/R AML patients [67].

### 2.4. TIM-3

T cell immunoglobulin and mucin domain 3 (TIM-3) is a suitable target for AML immunotherapy because is highly expressed on AML blasts and leukemic stem cells (LSCs) in most AML subtypes regardless of the patient’s genetic characteristics and treatment course [68]. TIM-3, a member of the TIM family, is a receptor expressed on IFN-γ-producing CD4^+^ and CD8^+^ T cells, identified as a co-inhibitory or checkpoint receptor or co-stimulatory receptor; co-blockade of TIM-3 and programmed cell death 1 (PD1) can result in tumor regression in preclinical models and can improve anticancer responses in patients with advanced cancers [68].

Several studies have explored anti-TIM-3 antibodies in combination with azacitidine in AML patients and in high-risk myelodysplasia (MDS) patients. A single-arm phase Ib trial evaluated the safety and the efficacy of Santolimab (anti-TIM-3 antibody) with azacitidine in 53 patients with high-risk MDS and 48 patients with newly diagnosed AML; among AML patients, 30% CR + Cri responses were observed, with a duration of response of 12.6 months in patients with a high-risk mutational profile (*TP53, RUNX1*, and *ASXL1* mutations) [69]. The randomized phase II study STIMULUS evaluated azacitidine plus Sabatolimab or azacitidine with a placebo in high-risk MDS patients: however, the primary endpoint of this study was not reached, for that concerns OS and PFS [70]. A phase II clinical study confirmed the negative results of this study, showing a PFS of 11.1 months in the Sabatolimab group compared to 8.5 months in the placebo group [71].

Zeiser and coworkers reported the results of the phase Ib/II multicenter study STIMULUS-AML2 (NCT 04623216) involving the treatment with Sabatolimab alone or in combination with azacitidine in AML patients who are in complete remission with positive MRD after allo-HSCT; the preliminary data on the 21 first patients enrolled in the safety run-in and treated with Sabatolimab alone either at 400mg or 800mg were recently presented [72]. Seven of the treated patients achieved a CR (3/10 at 400mg and 4/11 at 800 mg); four serious adverse events were observed, two at 400 mg and two at 800 mg [72].

Preclinical studies have shown the development of CAR-T cells engineered to efficiently target TIM-3-positive AML cells. Lee et al. have generated a CAR-expressing second-generation anti-TIM-3 antibody; CAR-T cells generated with this CAR exhibited potent antileukemic activity against AML cell lines, primary AML blasts, and human AML models in mice; furthermore, these CAR-T cells exhibited efficient killing of LSCs isolated from primary AML samples [73].

More recent studies have reported the development of bispecific TIM3-CD33 CAR-T cells: Particularly, anti-TIM-3 and anti-CD33 scFv sequences and co-stimulation domains have been cloned in a retroviral vector; CAR-T cells engineered with this CAR displayed an enhanced cytotoxicity against primary AML cells compared to monospecific CAR-T cells [74]. Among the various CAR constructs assayed, only split CAR-T constructs exhibited specific killing of CD33^+^TIM-3^+^ leukemic cells, sparing normal hematopoietic stem/progenitor cells [74]. These observations support the clinical evaluation of CD33/TIM-3 dual CAR-T cells [74].

### 2.5. CD123

CD123 is the alpha chain of the interleukin-3 receptor (IL-3 R) and is expressed at high levels in about 80% of AML and MDS [55,56]. CD123 expression is higher on leukemic cells than on normal progenitor and stem cells; furthermore, CD123 is a marker of leukemic stem cells, the cells that initiate and maintain the leukemic process [75,76].

For these reasons, CD123 is an attractive therapeutic target in AML and in other hematologic malignancies [77]. Various drugs targeting CD123 have been developed and evaluated at the clinical level: IL3 conjugated with diphtheria toxin; naked neutralizing anti-CD123 antibodies; drug-antibody conjugates; bispecific antibodies targeting both CD123 and CD3; chimeric antigen receptor (CAR) T cells engineered to target CD123 [77].

CD123 seems to be a suitable therapeutic target for some hematologic malignancies. In fact, Tagraxofusp, a fusion protein composed of IL3 fused with diphtheria toxin showed a marked antileukemic activity in blastic plasmocytoid neoplasia (BPDCN), a rare hematologic malignancy characterized by the proliferation of leukemic blasts with unique phenotypic properties, including also a particularly elevated expression of CD123; Tagraxofusp was approved by the FDA for the treatment of BPCDN on the basis of a clinical study showing 72% of CR rate in previously untreated BPCDN patients with a survival rate of 52% at 24 months [78,79]. However, the results observed in R/R AML patients were markedly less pronounced, with only one CR and two PR observed in 45 treated patients with a single infusion of Tagraxofusp [78]. A recent study reported the results of a phase I clinical trial exploring the safety and the efficacy of the combination of Tagraxofusp with azacitidine and Venetoclax in older AML patients; particularly, in a group of 26 older AML patients (median age 71 years) with previously untreated high-risk AML, 39% achieved a CR, 19% with incomplete count recovery and 12% with morphologic leukemia-free state [80]. Importantly, among 13 patients with *tp53*-mutated AML, 7/13 achieved a CR/Cri/MMFS, and 12/17 responders had no measurable residual disease, as assessed by flow cytometry; the mOS and mPFS were 14 months and 8.5 months, respectively [80].

Several BiTE targeting CD123 have been developed, and their therapeutic potential was explored in AML patients.

The MGD 066 BiTE was developed by MacroGenics (Rockville, MD, USA) and introduced with the commercial name of Flotetuzumab; MGD 066 is a CD3xCD123 DARt (dual-affinity re-targeting) protein composed of anti-human CD123Fv and humanized mouse anti-human CD3. Two phase I/II clinical studies have shown antileukemic activity of Flotetuzumab in R/R AML patients [81,82,83,84]. Particularly, a phase I study enrolling 30 R/R AML patients showed 19% of CR (31% among patients with refractory disease and 0% among those with relapsing disease) [81]. A phase I/II study enrolled 88 R/R AML patients and reported 26.7% of complete remission/complete remission with incomplete hematological recovery and an OS of 10.2 months among patients who achieved a CR [82]. The study of immune activation status in these patients provided evidence that an immune-associated gene signature could correlate with a response to Flotetuzumab [83,84]. Interestingly, Vadakekolathu and coworkers have reported a remarkable rate of response among patients with *TP53*-mutated AMLs: *TP53* mutant patients responsive to Flotetuzumab displayed a markedly higher tumor inflammation signature, inflammatory cytokines, FOXP3, CD8, and PD1 gene expression scores before therapy than patients non-responsive to treatment [14,85,86]. The analysis of the cellular neighborhoods of *TP53*-mutated using spatially resolved transcriptomics further supported the unique properties of *TP53* mutant AMLs compared to *TP53* WT AMLs, represented by enrichment of T cell-specific, type I/II IFN and proinflammatory genes, as well as leukemia stem cell programs: T cell dysfunctionality states and immune suppression [87]. From these observations, using a machine learning approach, a *TP53*-m-related “*spatial-metagene*” signature predicted poor prognosis in AML samples [87].

Other studies suggested that CD123 could represent a suitable therapeutic target for *TP53*-mut AMLs. Thus, Daver et al. have evaluated the safety and efficacy of a triple-drug combination based on azacitidine, Venetoclax, and Pivekimab Sunirine (PVEK, IMGN632), a CD123-targeting antibody conjugate in 50 newly diagnosed, older, and predominantly adverse-risk (38% *TP53*-mutant) AML patients [88]. In the whole population of AML patients, a CR + CRi of 66% was observed and in *TP53*-mutant AMLs of 47%; a total of 40% of the *TP53*-mutant AMLs with CR achieved an MRD-negative status compared to 73% observed in the whole AML population [88]. It is of interest to note that in the VIALE-A trial on elderly AML patients, 19.6% of *TP53*-mutant AMLs achieved a CR + CRi, and 30% of these patients had MRD-negativity [89]. The rate of MRD-negativity among patients achieving a CR was high in all molecular subgroups, including *FLT3, NPM1,* and *IDH*-mutant AMLs [88]. The majority of responding patients achieved early and deep remissions, which may translate to improved outcomes. However, the rate of responses achieved by PVEK administered as a single therapy in R/R AML patients was low, with an ORR of 21% and a composite complete remission rate of 17% [90].

Flotetuzumab was discontinued by the MacroGenics Company secondary to issues around dose administration and replaced by a second-generation agent called MGD-024. MGD-024 is a CD123xCD3 DART molecule designed for improved half-life, allowing its intermittent delivery; importantly, this molecule is engineered with a CD3-binding domain exhibiting reduced affinity for T cells and thus inducing a lower cytokine release [91]. These observations have supported the development of a phase I clinical study exploring the safety and efficacy of R/R AMLs [92].

XmAb1405 (with the commercial name of Vibecotamab) is a bispecific antibody targeting both CD123 and CD3; at variance with other bifunctional molecules, Vibecotamab is a full-length immunoglobulin molecule designed for a long half-life and thus to be intermittently administered [93,94]. A classical phase I study evaluated the safety and the efficacy of Vibecotamab and determined also the optimal dose for phase II clinical studies; this study enrolled a total of 120 patients with R/R hematological malignancies, including 118 AML patients [94]. In 16 of 120 patients, a dose-limiting event was observed; CRS was the most frequent event, but usually of grade 2 or less [95]. The optimal regimen for phase II studies consisted of a dosing regimen with three step-up doses in the first week (0.43, 0.75, 11.1 μg/Kg), followed by weekly dosing at 1.7 μg/Kg [95]. A total of 9% of patients achieved a CR; the rate of CRs was 39% for patients treated at the optimal dosage; the response was associated with lower baseline blast counts in blood and bone marrow [95]. A phase II study evaluated Vibecotamab after failure of treatment with hypomethylating agents in MDS patients and in MRD-positive AML patients [95]. The preliminary results observed in 12 AML patients, all pre-treated, including also Venetoclax treatment, showed that three patients were MRD-negative after treatment, while nine remained MRD-positive; the baseline mean MRD positivity in the patients responding to the treatment was clearly lower than that observed in non-responders (0.2% vs. 1.8%, respectively) [96].

APV0436 is a humanized BiTE targeting both CD123 and CD3; it is composed of two binding domains (fully human scFv binding to human CD123 and humanized scFv binding to human CD3) linked to a human IgG1 Fc domain (engineered to minimize complement fixation and interaction with Fcγ receptors) [97]. A phase Ib study defined the optimal dose for additional studies (0.2 μg/Kg) [98], and the expansion phase of this study provided preliminary data on the efficacy, with an objective response rate ranging from 20% in monotherapy to 33–40% in association with chemotherapy or Venetoclax, respectively [99].

Recent studies have evaluated trispecific molecules with the features of trifunctional natural killer cell engagers. The trifunctional natural killer cell engager (NKCE) SAR443579, targeting CD123 on leukemic cells and CD16a and NKp46 on NK cells, was recently developed; preclinical studies have shown the potent activity of this compound against CD123^+^ AML leukemic blasts, the promotion of NK cell activation, and induction of cytokine release in the presence of AML cells [100]. Based on this preclinical evidence, a phase I study was proposed to evaluate the safety and efficacy of SAR443579 in R/R AML and B-ALL patients [99]. The first report on this clinical study evaluated 23 R/R AML patients treated at six different dosage levels; treatment-related adverse events were reported in 16 patients, with no-dose limiting toxicities; the CR rate was 13%, with 37.5% of patients achieving a CR in the group treated at the highest dose [100]. A second report on this study included 42 R/R AML patients, showing a CR rate of 12% and 33% of CRs among patients treated at 1000 μg/Kg dose level: The median duration of CR is not estimable [101].

Other recent studies have reported the preclinical evaluation of trifunctional cell engagers targeting CD123 [102]. Thus, Gauthier et al. reported the development and characterization of a trifunctional NK cell engager targeting CD123 on tumor cells and CD16a and NKp46 on NK cells; this trifunctional antibody exerts a marked antileukemic effect in various leukemic models and exhibits the important property to bypass the resistance of AML blasts expressing CD64 to conventional anti-CD123 antibodies [102].

Few studies have explored the safety and efficacy of CAR-T cells targeting CD123. The first-in-human CAR-T 123 therapy clinical trial (NCT02159495) reported initial results in seven R/R AML patients, and three of these patients achieved either CR (1 patient) or morphologic leukemic-free state (MLFS, two patients) [103]. No patient developed treatment-related grade 3–4 adverse events [102]. Other studies were made using universal CAR-T (UCAR-T) prepared using T cells from healthy volunteers rather than those from patients. AVC-101 (UCAR-T cells) is an adaptor CAR-T, consisting of a universal CAR-T cell and a CD123 targeting module™; the TM binds to the CAR-T cell via a peptide TAG and induces T cell activity against CD123 bearing cells; since the TM had a short half-life, interruption of its continuous administration rapidly switches T cell activity off, thus mitigating toxicities [104]. A total of 19 patients were enrolled, 15 with R/R AML and 4 with CR MRD-positive AML, heavily pre-treated, and 12 with prior allo-HSCT; ORR was 53% in R/R AML patients and 75% in CR, MRD-positive patients; the introduction of an expanded TM administration resulted in more durable responses, and one patient with MRTD-positive AML had a strong reduction in MRD positivity and became transplant eligible [104].

### 2.6. Targeting of NKG2D in AML

CYAD-01 is an autologous CAR-T cell product based on the NK group 2D (NKG2D) receptor that binds the eight ligands that are overexpressed in a wide range of hematological malignancies but are absent on non-neoplastic cells [105]. The multicenter THINK study (an open-label, dose-escalation, phase I study, NCT03018405) evaluated the safety and the antileukemic activity of CYAD-01 in AML, MD, and MM patients at three dose levels: 3 × 10^8^ (dose level 1), 1 × 10^9^ (dose level 2), and 3 × 10^9^ (dose level 3) [105]. A total of 16 patients were treated with CYAD-01, including 3 patients at dose 1, 3 patients at dose 2, and 6 patients at dose 3 [104]. A total of 44% of treated patients had grade 3 or 4 treatment-related adverse events, and 31% of patients had grade 3 or 4 CRS; one dose-limiting toxicity was observed at dose level 3 [104]. A total of 25% of the patients had an objective response; two of these responding patients proceeded to AHSCT with durable remissions (up to 61 months) [105]. Further clinical studies with NKG2D ligand by CAR-T cell therapy are warranted [105].

Attempts to improve the efficacy of CYAD-01 could consist of incorporating lymphodepletion in the CAR-T cell protocol. Furthermore, a second-generation CAR-T cell product, CYAD-01, would abrogate potential in vivo fratricide effects through CAR-T cell upregulation of NKG2D ligands.

However, a limitation of CYAD-01-mediated therapy seems to be related to the absence of NKG2D ligands on LSCs; this limitation could be bypassed using therapy in association with agents such as PARP1 inhibitors that upregulate NKG2D ligand expression on LSCs [106].

The second-generation CAR-T product CYAD-02 was used in a phase I clinical trial (NCT 04167696): Of the 11 patients enrolled with R/R AML or MDS, 2 patients with MDS achieved marrow CR, while no patients with MAL achieved CR [107].

A recent study reported the development and characterization of dual-targeted CAR-T (123NL CAR-T) cells targeting both CD123 and NKG2DL; 123NL CAR-T cells eradicate AML cells and target also immunosuppressive cells [108]. A marker/suicide gene, ROR8, which binds targeting epitopes of CD34 and CD20 antigens, was incorporated into the CAR structure, thus allowing the elimination of 123NL CAR-T cells and cessation of their cytotoxicity through infusion of Rituximab [108].

Recently, a phase I study reported the evaluation of NKX101, a CAR-NK cell therapy based on NK cells derived from healthy donors engineered to express an NKG2D ligand-directed CAR and a membrane-bound form of IL-15 to promote persistence and activity of CAR-T cells; fludarabine/Ara-C was used as an alternative to fludarabine/cyclophosphamide for lymphodepletion [109]. Six R/R AML patients were explored in this phase I study; four of six patients had CR/Cri, with three in CR: Three of these responders were MRD-negative by flow cytometry; one patient in CR was bridged to allo-HSCT and remained in remission [109]. Pharmacokinetic studies showed that NKX101, despite being an allogeneic product, persisted for up to three weeks [109]. The toxicity profile was manageable and mainly related to the AML condition and to the lymphodepletion regimes, with events of CRS or ICANS [109].

### 2.7. Other Membrane Antigens Targetable in AML Patients

Other membrane antigens have been identified as possible targets for immunotherapy of AML patients.

CD93 is a cell surface lectin that has a role in both cell–cell adhesion and host immune defense and is highly expressed at diagnosis and at relapse in AML in 50% of cases [110]. Furthermore, CD93 was identified as a marker of a cycling, non-quiescent leukemia stem cell population in MLL-rearranged AML [111]. For this profile of expression on leukemic cells and for its absent expression on nonhematopoietic tissues, CD93 appears to be a suitable marker for AML immunotherapy. However, CD93 cells resulted to be expressed on normal endothelial cells, and canonical CD93-directed CAR-T cells kill human endothelial cells [111]. To bypass the problem of shared antigen expression between AML and endothelial cells, Richards and coworkers generated CD93-targeting CAR-T cells with a NOT-gated strategy that limits the CAR-T cell killing of endothelial cells [112]. There are no clinical trials involving CD93 targeting in AML.

CD70 is the natural ligand for CD27, a member of the TNF superfamily. CD70 is heterogeneously expressed on AML primary cells, including leukemic blasts, leukemic progenitor, and leukemic stem cells, but not expressed on normal hematopoietic stem cells and on the majority of blood cells [113]. LSCs upregulate CD70 expression in response to treatment with hypomethylating agents; preclinical studies showed that blocking CD70/CD27 signaling with the anti-human CD70 antibody Custazumab resulted in elimination of LSCs in vitro and in xenotransplantation experiments [113]. Based on this evidence, a phase I/II clinical trial was performed in older untreated AML patients with a single dose of Cusatuzumab, followed by combination therapy with azacitidine; a total of 8/12 treated patients achieved a CR, with 4 patients achieving MRD negativity by flow cytometry at <10^−3^ [114]. Phase II of this study enrolled 26 AML patients, 21 with adverse risk: An objective response was observed in 8/26; at a median follow-up of 10.9 months, the mOS was 11.5 months [115]. Several groups have reported the development of CD70-specific CAR-T cells exhibiting potent activity against AML cells, with no toxicity for normal HSCs [116,117]. Recently, Bianchi et al. reported the development of a multispecific T cell-engaging designed ankyrin repeat protein (DARPin), MP0533, designed to bind CD3 on T cells while simultaneously binding CD33, CD123, and CD70; the optimal binding affinity of MP0533 to each leukemia antigen is intended to enable an activity-like selectively and efficacy window to preferentially kill AML cells co-expressing at least two leukemic antigens while sparing single-expressing healthy cells [118]. A phase I/IIA clinical study (NCT 05673057) was developed using MP0533 in relapsed/refractory AML patients. A preliminary report on this study showed a complete response in one of two treated patients; the responding patients displayed a *TP53*-mutant R/R AML [119].

The interleukin 1 receptor accessory protein (IL1RAP) is a member of the IL1 superfamily that recently emerged as a potential target for AML. IL1RAP is highly expressed on the membrane of AML and high-risk MDS [120]. Importantly, IL1RAP is expressed at significantly higher levels in LSCs than in normal HSCs, thus supporting that it may represent a potential immunotherapeutic target for AML [121]. To this end, BOS-371, a humanized monoclonal antibody anti-IL1RAP that has been Fc-engineered to enhance its ADCC and to deplete IL1RAP-expressing AML cells displayed potent antileukemic activity in various animal models [122]. IL1RAPmnCAR-T cells have been generated and showed antileukemia activity in preclinical models and are being tested in clinical trials [123]. A recent study reported the development of an IL1RAP-specific T cell engager (BIF002) showing a potent antileukemic activity and showing, in preclinical models, the clearing of the LSC fraction [124].

## 3. Reasons for Immunotherapy Failure for AML and Potential Strategies for Overcoming These Limitations

### 3.1. AML Blast Genetic and Epigenetic Heterogeneity and Multi-Target Therapy

The interindividual and intraindividual heterogeneity of genotypes and phenotypes in AML represents one of the major obstacles to the eradication of bulk AMLs and LSCs through immunotherapeutic targeting of membrane antigens preferentially expressed on leukemic cells. Future immunotherapeutic strategies attempting to eradicate AML cells through multiple antigenic targeting, eventually based on the individual phenotypic profile of each AML patient, could represent a valuable strategy to bypass this important limitation.

It is of crucial importance to better define the heterogeneity of the membrane antigen expression profile at the level of AML subsets and of the individual patient.

A remarkable example of the heterogeneity of AML is provided by the study of t(8;21) AMLs. AMLs with t(8;21) represent a molecular subtype of MALs corresponding to about 8% of AMLs and are characterized by the t(8;21) translocation generating the *RUNX1-RUNX1T1* fusion gene. In spite of this molecular homogeneity, t(;21) AMLs are heterogeneous in that 30% of patients relapse after standard therapy. A multidimensional study explored the origins of this heterogeneity and discovered that relapsing t(8;21) AMLs are characterized by the presence of a higher proportion of CD34^+^CD117^dim^ myeloblasts, with characteristics of GM-progenitors and drug resistance [125]. A subsequent integrative cell analysis showed that this immunophenotypic heterogeneity is associated with a concomitant immunologic heterogeneity in that t(8;21) AMLs with more abundant CD34^+^CD117^bright^ cells have a higher number of infiltrating CD8^+^ lymphocytes and overexpression of immune exhaustion-related genes [125]. Data integration analysis of single-cell dynamics showed that a subset of T cells (cluster-2) characterized by high expression of GZMB, NKG7, PRF1, and GNLY genes is markedly expanded in t(8;21) patients relapsing after therapy [126]. The T cell cluster-2 signature can be utilized to stratify patients by overall survival outcome [126].

Surface proteomic studies and single-cell RNA sequencing approaches hold great promise for membrane antigen identification in AML. Through the analysis of the surface proteome of 100 genetically different primary AML specimens, Bordeleau et al. identified numerous antigens and markers preferentially expressed at the level of primitive AML cells; interestingly, several antigens and markers are selectively or preferentially expressed by AML subgroups [127]. At the level of AML antigens expressed in most cases, in addition to known markers, other antigens were identified, including PTPRC, CD47, CD37, ITAG4, and CD74 [127]. Some membrane antigens, such as ADGRG1, CD7, and CD96, were preferentially expressed in some AML subtypes [127]. Importantly, many of the newly identified antigens are targeted by antibodies currently under clinical evaluation.

Other studies have shown that ADGRG1, also known as GPR56 (the adhesion protein-coupled receptor 56) is a marker of LSCs, and its expression on AML cells is significantly associated with the high-risk genetic subgroup and a poor outcome [128]. GPR56 expression was higher in the leukemic cell subpopulation CD34^+^CD38^−^, enriched in LSCs, compared to CD34^+^CD38^+^ or CD34^−^ cells; furthermore, in CD34^+^ AMLs, high GPR6 expression correlates with the presence of a leukemic stem cell signature [129]. Furthermore, AML patients with high expression of GPR56 at diagnosis have a higher risk of relapse after allo-HSCT [130]. Interestingly, the study of AML patients undergoing allo-HSCT showed that patients achieving a remission status display a CD8^+^ T-lymphocyte population more advanced in maturation and with a stronger cytotoxicity signature; these CD8^+^ lymphocytes were characterized by high GPR56 expression; in responding patients, the levels of GPR56 expression increase after allo-HSCT [131,132]. The results of these studies raise an important, unresolved question of whether GPR56 expression on CD8^+^ lymphocytes constitutes a simple marker or mechanism of effective graft versus leukemia allo-reactivity [133].

Haubner et al. have initially explored the profile of expression of six known LSC membrane markers in a large cohort of primary AMLs and reached the conclusion that the dual combinations of CLL1/TIM3 and CD33/TIM3 are highly positive in AMLs compared with normal hematopoiesis and non-hematopoietic tissues [134]. More recently, in an attempt to bypass these limitations, Haubner et al. have explored the profile of expression of membrane antigens on the leukemic blasts of 39 patients with R/R AML and observed that one of the top candidates with high expression in normal hematopoietic tissue and other tissues is the adhesion G protein-coupled receptor ADGRE2; the level of ADGRE2 expression on AML blasts correlates with a poor molecular risk profile and represents an independent prognostic factor [135]. Quantitative measurements of expression of various target antigens, including ADGRE2, CD38, CD123, and CLL1, showed that the combinational targeting of ADGRE2 and CLL1 allowed the efficient elimination of AML cells and of patient-derived xenograft models with limited toxicity for normal stem/progenitor cells [98]. Other studies have shown that ADGRE2 is expressed at high levels in all AML patients, while it is expressed in T cells in only a minority of AML patients [136]. Furthermore, a recent study provided evidence that ADGRE2 promotes LSC self-renewal and leukemogenesis by modulating proteostasis through a MEK/AP1/DUSP1 axis [137]. The combination of an attenuated ADGRE2-CAR with a CLL1 co-stimulating receptor (ADCLEC-synth1) allowed the preferential targeting of ADREG2^+^/CLL1^+^ LSCs over ADREG2^low^/CLL1^−^ normal stem/progenitor cells [137]. Preclinical studies have shown a higher efficacy of ADCLEC-synth 1 to induce complete remissions compared to CD33-CAR-T cells [137]. These studies have provided the basis for an ongoing clinical phase I study (NCT05748197) based on CAR-T cell engineering with ACDLEC-synth1 in R/R AML patients [138].

An alternative strategy for controllable multiplex leukemic targeting [adapter CAR (AdCAR) system] was developed by Seitz et al. and was based on the generation of AdCAR-T cells directed to surface antigens through biotin-labeled adapter molecules in the context of a specific linker structure, defined as Linker-Label-Epitope [139]. In a subsequent study, it was shown the feasibility of sequential application of adapter molecules of different specificities to target CD33, CD123, and CLL1 on leukemic cells; furthermore, this technology offers the opportunity to counteract T cell exhaustion related to chronic T cell stimulation through the introduction of treatment-free intervals [140]. The same authors have more recently supported the rational basis of combinatorial antigen targeting by adaptor CAR-T cells in pediatric AMLs [140]. In the first set of experiments, they showed that target antigens, including CD33, CD38, CD123, CD135, CD371 CD276, and IL1RAP, are heterogeneously expressed in 30 pediatric AMLs; both marked intertumoral and intratumoral heterogeneity was observed [141]. Only multiplex targeting by AdCAR-T cells was able to successfully address AML intratumoral heterogeneity and prevent antigen escape. These observations support clinical studies involving the evaluation of adopCAR-T cells in R/R AML patients.

Siddiqui et al. reported the development of a split universal, programmable (SUPRA) CAR platform, affording tunability over activation levels and multiplex targeting; particularly, experimental studies evaluated combined CD33 and FLT3 targeting, showing that the SUPRA CAR platform allowed the generation of CAR-T cells targeting both CD33 and FLT3 with equivalent efficiency compared to conventional CARs and allowed also to modulate their cytotoxic activity to minimize off-target cytotoxicity against normal HSCs and HPCs [142].

### 3.2. Limited Effector Function Through T Cell Intrinsic and Extrinsic (Immunosuppressive Microenvironment) Mechanisms

A major challenge in studies of immunotherapy in AML patients is seemingly related to the high level of T cell dysfunction observed in R/R AML patients. Recent studies have clearly shown reduced T cell fitness in AML patients at diagnosis or at relapse. CD8^+^ T cells may enter a senescent-like state, exhibiting impaired functionality in AML (impaired killing of AML blasts); the proportion of these senescent CD8^+^ T cells negatively correlates with overall survival [15]. Korecani and coworkers have explored T cell fitness during AML progression comparing longitudinally AML patients at diagnosis, remission, and relapse [143]. The percentage of bone marrow CD3^+^ T cells was lower at diagnosis and relapse compared to complete remission or healthy controls; terminally effector cells were the most abundant CD8^+^ population at diagnosis, central memory cells the most abundant CD8^+^ cell population at relapse, and naïve T cells at complete remission; AMLCD8^+^ lymphocytes showed a significantly higher expression of exhaustion-associated inhibitory receptors (CD244, PD1, TIM-3, CD160, LAG-3) compared to normal bone marrow controls, a higher frequency of CD4^+^ and CD8^+^ T cells co-expressing PD1, TIM-3, and LAG-3 at diagnosis and relapse compared to complete remission; functional T cell assays showed impaired function during active disease; after continuous BiTE stimulation, AML T cells at diagnosis and at relapse displayed decreased effector molecule production and impaired metabolic fitness in comparison to complete remission [143]. The impaired fitness of T cells during the active phases of AML, either at the time of diagnosis or relapse, and their functional repair at complete remission support the use of BiTE or CAR-T in patients at remission [143].

Another recent study by Desai and coworkers performed a single-cell RNA profiling of CD8^+^ T cells from AML patients at diagnosis and at relapse [143]. Through this analysis, they identified two effector CD8^+^ T cell subsets characterized by distinct cytokine and metabolic profiles that were differentially enriched in patients at diagnosis and at relapse [144]. CD8^+^ T cells from relapsed/refractory AML patients had a higher degree of clonal hyperexpression associated with terminal differentiation and with a higher CD8-derived signature correlated with poorer outcomes in untreated AML patients [143]. These findings support the conclusion that immunological response and reconstitution are likely to be most successful at earlier disease stages when CD8^+^ T cells are less differentiated and have greater capacity for clonotype transitions [144].

In another recent study, Mazziotta et al. provided a detailed characterization of CD8^+^ T cell populations in AML patients undergoing standard chemotherapy treatment at baseline and after treatment; patients achieving a complete response were classified as responders and the others not achieving a complete response as non-responders [145]. CD8^+^ lymphocytes were characterized using different approaches, such as spectral flow cytometry, bulk transcriptomics, and single-cell RNA sequencing in conjunction with T cell receptor sequencing [145]. In this study, CD8^+^ T cells were classified according to their differentiation stage into naïve, early memory, and terminally differentiated senescence-like cells [145]. The ratio of early memory vs. terminally differentiated CD8^+^ T cells was positively associated with response to AML induction chemotherapy and improved overall survival [145]. Seemingly, a higher number of early memory cells may underline the existence of an immune system with the potential of generating a leukemia-specific T cell response, while a higher number of terminally differentiated, senescence-like CD8^+^ T cells may underline a prior chronic response to AML-specific antigens to generate a T cell-mediated antileukemia response [145].

All these observations support the existence of T cells with low fitness in AML patients with active disease. Thus, these studies support the development of immunotherapy studies in AML patients in remission. This conclusion is further supported by various observations made in AML immunotherapy studies. Thus, Ravandi et al., using the CD123-CD3 bispecific antibody Vibecotamab in R/R AML patients, showed the existence of a strong association between low blast cell counts (<20%) and complete response to immunotherapy [94,95]. These observations support future clinical trials of immunotherapy in R/R AML patients with a low tumor burden or in AML patients in complete remission. Another observation supporting this view was made in a clinical trial based on the administration of azacitidine and Nivolumab to R/R AML patients, showing an ORR of 32%, compared to 20% observed in historical controls on comparable patients treated with hypomethylating agents; only a few patients displayed long-term remissions [146]. Pre-therapy bone marrow and peripheral blood CD3^+^ and CD8^+^ T cell levels were significantly predictive for response [146]. Importantly, responses were observed in patients who had received only one or two lines of prior salvage therapy, thus suggesting the degree of T cell dysfunction associated with AML progression and/or with the number of prior therapies and negatively affecting the response to immunotherapy [146].

### 3.3. Minimizing On-Target/Off-Target Toxicities of AML Immunotherapies

AML immunotherapies target genes expressed also by normal HSCs/HPCs, often resulting in on-target/off-tumor toxicity. A recent study reported the development of an epitope editing approach to engineer donor HSCs/HPCs used for bone marrow transplantation, generating in these cells a mechanism of selective resistance to CAR-T cells or to antibodies [106]. Through epitope mapping and library screenings, amino acid changes abrogating the binding of therapeutic antibodies targeting CD123, FLT3, and KIT were identified; using this base editing approach, these modifications have been introduced into CD34^+^ HSCs/HPCs that retain engrafting and long-term repopulating capacities [147]. Epitope-modified hematopoiesis was resistant to the toxic effects of CAR-T cell therapy [147].

To minimize the off-tumor toxicity of CAR-T cells, switchable CAR-T cells have been developed. Off-switch strategies involve the use of molecules or drugs that hamper the function of CAR-T cells in a timely manner. The suicide gene strategy involves the transduction of apoptotic genes into cellular therapeutic products to switch off CAR-T cell activity in a timely manner. A notable example is given by UCART123 cells expressing RQR8 that allow their elimination with Rituximab administration [148]. On-switch strategies involve the development of CAR-T cells whose activity is modulated by targeting modules (TM). One example is given by the UniCAR system in which there is a CAR for an inert manipulation of T cells and specific TMs for redirecting UniCAR-T cells in an individualized target- and time-dependent manner [149]. In this system, off-tumor toxicity can be controlled by suspending the administration of TM [149].

### 3.4. Multiple AML Targeting Involving Membrane Antigens and Oncogenic Proteins

A recent study proposed a double targeting immunotherapy strategy with one target represented by mutant oncogenic protein (NPM1) and the other target represented by CD33. Patients with mutant *NPM1* (*ΔNPM1*) bear a characteristic 4b frameshift insertion in exon 12 of the *NPM1* gene; the resulting NPM1 mutated protein is 4 amino acids longer than WT *NPM1,* and its C-terminal 11 amino acids are translated in an alternative reading frame (CLAVEEVSLRK). Through screening of the HLA class I ligandome of primary *NPM1*-mutant AMLs, CD8^+^ T cells with reactivity against AML cells were isolated; from one of these clones, the T cell receptor was isolated and its retroviral transfer to CD8^+^ and CD4^+^ cells conferred specific recognition and lysis of *NPM1*-mutant AML cells [150]. Based on these observations, Teppert and coworkers have developed a double-targeting immunotherapy combining CAR and TCR technologies: CAR’TCR-T cells, co-expressing CD33-CAR and a transgenic dNPM1-TCR, showed potent and prolonged anti-tumor activity against *NPM1*-mutant AMLs [151].

### 3.5. AML Immunotherapy Post-Transplantation

It is well-known that allo-HSCT is the only potentially curative therapy for patients with high-risk AML; however, relapse occurs in about 30–40% of patients undergoing allo-HSCT. Therefore, there is an absolute need to evaluate new therapies that post-transplantation may decrease the risk of relapse and improve survival. In this context, Shah and coworkers have proposed a phase I/II study aiming to evaluate the safety and efficacy of the infusion of VCAR33, donor-derived CAR-T cells targeting CD33 [152]. The trial will enroll two arms of patients subdivided according to their AML disease burden: arm A leukemic blasts ≥ 5%; arm B leukemic blasts ≤ 5% in the bone marrow [152]. A long-term follow-up of patients, up to 15 years, is planned [152].

### 3.6. Cytokine-Mediated CAR-T Therapy Resistance in AML

A recent study reported a novel mechanism responsible for CAR-T cell resistance in AML. Bhagwat and coworkers reported the results of a study of autologous anti-CD123 CAR-T cell therapy in 12 adult patients with R/R AML; three of these patients achieved a response to treatment (two complete responses molecular MRD-negative and one CR with incomplete hematologic recovery) [153]. Cytokine release syndrome was observed in 10/12 treated patients [153]. The occurrence of very frequent CRS in these patients suggests CD123-CAR-T cells are biologically active also in patients not achieving a clinical response following CAR-T cell infusions. To explore the possible mechanisms mediating resistance to CD123-CAR-T cells, these authors characterized the cytokines released during CAR-T cell therapy and observed the production of cytokines such as IL-6, IFN-γ, and G-CSF that promote CAR-T cell viability but do not affect AML cell viability but also other cytokines such as GM-CSF, IL-3, and FLT3L that markedly increase AML cell viability and proliferation [152]. Cytokines such as IL-3, GM-CSF, and FLT3L protect AML cells from the cytotoxicity mediated by CAR-T cells, as well as the serum of patients treated with CAR-T cells obtained in correspondence of in vivo peak of CAR-T cells [152]. Parallel studies performed in B-ALL patients undergoing CAR-T cell therapy showed that this mechanism of cytokine-mediated prosurvival effect is unique to AML cells [151]. Extended exposure of AML cells to CAR-T cells caused T cell exhaustion that can be restored through the addition of JAK/STAT or BCL2 inhibitors [153]. In conclusion, this study supports the view that CAR-T-CD123 immunotherapy in AML patients may be undermined by the cytokines induced by this therapy and its outcome could be improved by concomitant addition of JAK/STAT or BCL2 inhibitors [153].

## 4. Conclusions

At variance with other hematological malignancies, such as lymphoblastic acute leukemia, lymphoma, and myeloma, for which several therapeutic antibodies have been approved for clinical use, the development of therapeutic antibodies in AML progressed more slowly. These difficulties are mainly related to the consistent interindividual and intraindividual genetic and phenotypic heterogeneity of AMLs.

At present, there are no commercially available CAR-T cell products for the therapy of AML patients. Proof-of-principle has been provided about the clinical efficacy of some of these CAR-T cell products in a part of AML patients. However, the available clinical results are limited; there is no clear evidence about the optimal targets, and toxicity issues remain unresolved. Particularly, the preclinical and clinical studies carried out using CAR-T cells in AML patients have shown the existence of several major obstacles mainly represented by the following: the identification of target antigens that are expressed on leukemic cells but absent or expressed at very low levels in normal hematopoietic cells (including HSCs/HPCs); intertumoral and intratumoral heterogeneity of expression of current AML antigens targeted in immunotherapy studies; the optimal cell source to be used for CAR-T cell generation; the existence of intrinsic and extrinsic mechanisms limiting the effector function of CAR-T cells. These obstacles may be in part bypassed by targeting more than one antigen on AML cells. Among the various antigens targeted by CAR-T cells in AML cells, CLL1 provided the most promising results.

Studies on therapeutic antibodies targeting AML cells were focused on the development of bispecific antibodies, particularly of bispecific immune cell engagers. Identifying the most appropriate target antigen was a major challenge for the successful development of BisAbs for AML therapy. However, in spite of the limitations, studies carried out using BisAbs targeting CD123 have provided evidence that *TP53*-mutant AML may represent an important target. In fact, this AML subtype seems to be more responsive to immunotherapy than to standard-of-care cytotoxic chemotherapy.

As outlined in the section on future perspectives, several ongoing studies are evaluating or will evaluate new strategies to improve the efficacy and limit the toxicity of immunotherapies in AML patients, and there is a reasonable hope that these studies will contribute to improve the outcomes of these patients.

In conclusion, immunotherapies for AML face significant challenges but several promising strategies are in development.

## Figures and Tables

**Table 1:** Main cytogenetic abnormalities observed in AML.

Risk Category	Cytogenetic Abnormality
Favorable	t(15;17) PML-RARA t(8;21) RUNX1-RUNX1T1 Inv(16) or t(16;16) CBFB-MYH11
Intermediate	Trisomy 8 alone t(9;11) MLLT3-KMT2A
Adverse	t(6;9) DEK-NUP214 t(v;11q23.3) KMT2A-rearranged t(9;22) BCR-ABL1 t(3;3) MECOM Monosomal karyotype (-5, 5q^-^, -7, 7q^-^, -17)/abnormality (17p) Inv(3) Complex karyotype (≥3 clonal chromosomal abnormalities)

**Table 2:** AML target antigens used in clinical studies.

Target Antigen	Physiological Role	Expression on Bulk AML Cells	Expression on LSCs	Expression on Normal HSC/HPC	Expression in Non- Hematopoietic Tissues	Immunotherapy Applications
CD33 (Siglec-3)	Sialic acid-dependent cell interactions and adhesion of myeloid cells	++/+++ (95%)	+ (88%)	+/++	Kupffer cells, microglia	Abs, BiTEs, trispecific Abs, CAR-T, bispecific CAR-T
CLL1 (C-type lectin-like molecule 1)	Inhibitory lectin-like receptor. Immune regulation as an inhibitory receptor	++/+++ (80%)	++ (45%)	−/− Expression on few lymphoid progenitors	Absent	Abs, BiTEs, trispecific Abs, CAR-T, bispecific CAR-T
TIM-.3 (T cell immunoglobulin and mucin domain 3)	Immunoregulatory protein (it inhibits excessive or prolonged immune activation)	++ (87%)	++ (78%)	−/+	Absent	Abs, CAR-T
CD123	Interleukin-3 receptor alpha chain	++/+++ 96% Overexpressed in 60–70% of cases	++ (95)	+/+	Endothelial cells	Abs, BiTEs, trispecific Abs, CAR-T, bispecific CAR-T, NK-CAR
CD38	Adhesion partner for CD31. Enzymatic activity (NAD and NADP catabolism)	+++ (80–90%)	Low +	−/++	Mature hematopoietic cells	Abs, BiTEs, trispecific Abs, CAR-T
ADGRE2 (Adhesion G Protein-Coupled Receptor 2)	Cell membrane receptor that binds to glycosaminoglycan chains and promotes cell attachment	++/+++ (82%)	++/+++ (82%)	+/+	Absent	ADCLEC-syn1 CAR-T

**Table 3:** Main clinical studies involving the use of CAR-T cells targeting CD33.

Clinical Trial	Patient Number (Age)	Source	Co-Stimulatory Domain	Safety	Efficacy
NCT 031268649	10 (18–73 yr) Only 3 patients infused	Autologous	4-1BB	CRS 66% ICANs 33%	No response
NCT 03971799	19 DL1 3 × 10^5^/Kg DL2 1 × 10^6^/Kg DL3 3 × 10^6^/Kg DL4 1 × 10^7^/Kg (18–73 yr)	Autologous	CD28	CRS 68% CRS3 21%	2 CRs at DL4 Optimal CAR-T cell expansion at DL4
NCT 0485519	4	Autologous	Not reported	CRS 1–2 75% CRS 4 25% ICANS1 50%	Cri, MRD^−^ 50%
NCT 03927261	11	Autologous	Second generation Not reported	CRS 69% CRS3 6% ICANS 6%	CR + CRi 3pts
NCT 038335519	4 (3–12 yr) CAR-T cells overexpressing c-JUN	Autologous	CD28	CRS 1–2 75% CRS4 25% ICANS 50%	Cri, MRD^−^ 50% PR 25%
NCT 04849910 VBP01	6	Autologous	Not reported	Not reported	Positive engraftment of allo-HSCT
CD33 CAR-NK cell therapy	10 (10–65 yr)	Autologous	Not reported	CRS2 10%	CD, MRD^−^ 60%

**Table 4:** Main clinical trials involving the use of CAR-T cells targeting CLL1.

Clinical Trial	Patient Number (Age)	Source	Co-Stimulatory Domain	Safety	Efficacy
NCT 093222674	7 (6–12 yrs)	Autologous	CD28/CD27 (4pt) 4-1BB (3pt)	CD28/CD27: CRS1 75% CRS2 25% ICANS 0% 4-11B: CRS1 66% CRS2 33% ICANS 33%	CD28/CD27: PR 25% CR, MRD^−^ 75% 4-1BB: PR 33% CR MRD^+^ 33% CR MRD^−^ 33%
Not reported Zhang et al. [62]	8	Autologous	4-1BB	CRS 1–2 100%	MLFS MRD^−^ 50% MLFS MRD^+^ 12.5% CRi MRD^+^ 12.5% PR 12.5% SD. 12.5%
Chi CTR20000041054	10 (18–73 yrs)	Autologous	4-1BB	CRS 100% CRS3 60% ICANS 0%	CRi 70% (6/7 pts in CRi allo-HSCT)
Chi CTR20000041054	30 (18–73 yrs) DL1 0.5 × 10^6^/Kg DL2 1 × 10^6^/Kg DL3 1.5 × 10^6^/Kg DL4 2 × 10^6^/Kg	Autologous	4-1BB	CRS 1–2 60% CRS3 36.7% CRS4: 3.3%	CR + Cri DL1 44.4% DL2 55.5% DL3 50% DL4 0%
NCT03018405	9	Autologous	Not defined	CRS 89% ICANS 44%	CR MRD^−^ 7/9 pt
